# Retrieval of Embolized Amplatzer Patent Foramen Ovale Occlusion Device: Issues Related to Late Recognition

**DOI:** 10.1155/2017/9894215

**Published:** 2017-04-16

**Authors:** Allan J. Davies, Nicholas Collins, Nicole Organ

**Affiliations:** ^1^Cardiovascular Department, John Hunter Hospital, Newcastle, NSW, Australia; ^2^Department of Vascular Surgery, John Hunter Hospital, Newcastle, NSW, Australia

## Abstract

Embolization of a percutaneous patent foramen ovale (PFO) closure device is a rare but serious complication. While early, periprocedural device embolization can normally be managed with snare and percutaneous retrieval, late embolization requires a different management strategy due to inability of the device to deform to allow passage into a large caliber sheath. We present a case of asymptomatic device embolization recognized six months following implantation and discuss the challenges encountered in successfully retrieving the device.

## 1. Introduction

Percutaneous device closure of patent foramen ovale (PFO) has been utilized as a therapy for the prevention of recurrent cryptogenic stroke. With appropriate patient selection, low rates of recurrent embolic events can be achieved, with accrual of benefit seen with long-term follow-up [1, 2]. Procedural complications are uncommon, with vascular injury [3], cardiac perforation [4], air embolism, device fracture [5], and cardiac arrhythmia [6] reported. Device embolization is uncommon in PFO closure, particularly when compared to device closure of atrial septal defects. When device embolization occurs, it is typically recognized early, with delayed or late retrieval uncommon.

We describe the late retrieval of an asymptomatic embolized patent foramen ovale occlusion device 6 months following device implant. The complicating issues relevant to late retrieval are discussed including the lack of deformability of the device and risk of distal embolization when difficulty with percutaneous retrieval is encountered.

## 2. Case Report

A 58-year-old man was referred for patent foramen ovale closure following a presumed cryptogenic stroke. The patient presented with transient hemiparesis, with MR imaging suggesting a previous additional, unrecognized cerebellar infarction. CT angiography of the neck and intracranial vessels was unremarkable. Ambulatory ECG monitoring did not detect any atrial fibrillation. Contrast transthoracic echocardiography suggested the presence of a patent foramen ovale with subsequent transoesophageal echocardiography confirming the presence of a PFO with an aneurysmal interatrial septum. In view of the absence of alternative mechanisms of stroke, MR imaging suggesting previous embolic events, and the desire to avoid lifelong anticoagulation, the patient was referred for percutaneous device closure.

**Figure 1:**
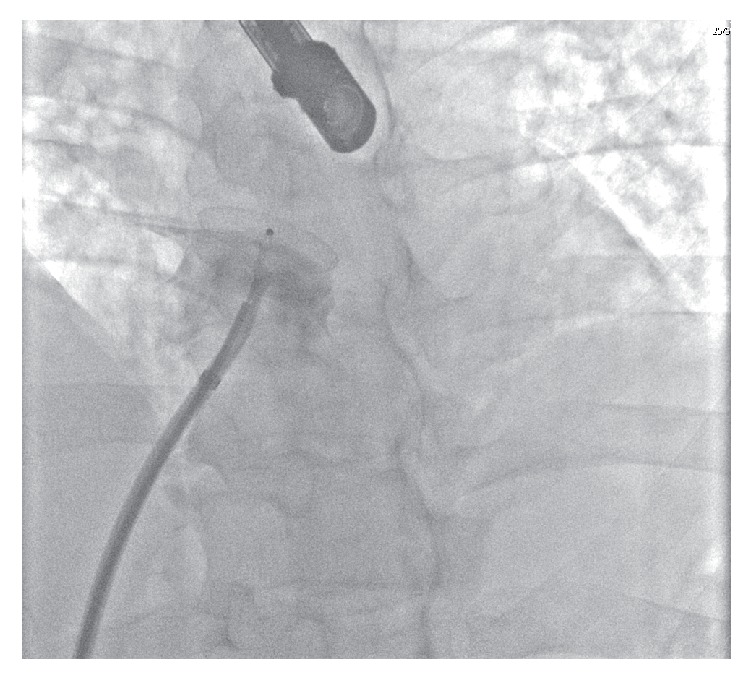
Right atrial angiography demonstrating patent foramen ovale closure device position prior to device release.

**Figure 2:**
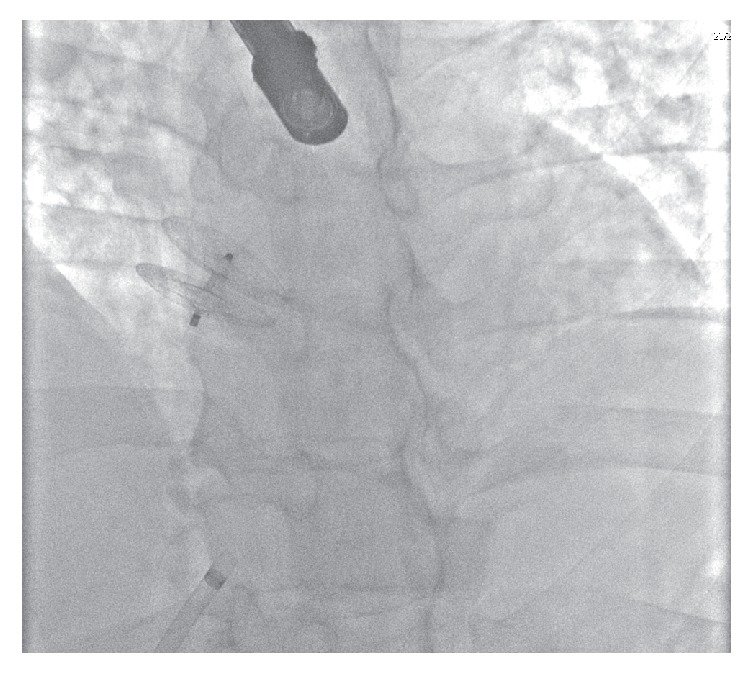
Patent foramen ovale occlusion device positioned appropriately across atrial septum after release.

Under general anaesthesia and transoesophageal echocardiography guidance, a 30 mm Amplatzer fenestrated atrial septal occluder was delivered using a 9 Fr delivery system. The device was released uneventfully (Figure 1) and the final appearance on fluoroscopy (Figure 2) and transoesophageal echocardiography was excellent; device position was confirmed with a “push-pull” maneuver prior to device release. A transthoracic echocardiogram was performed the following day demonstrating satisfactory device position with no evidence of pericardial effusion. The patient was then discharged on aspirin and clopidogrel. The patient remained asymptomatic. Routine repeat transthoracic echocardiography was undertaken six months following device implantation to assess for any residual shunt. The Amplatzer occlusion device was not visualized on transthoracic imaging with strongly positive right to left shunt noted. Subsequent transoesophageal imaging demonstrated absence of the occluder across the interatrial septum consistent with device embolization; the device could not be visualized in the main or branch pulmonary arteries. CT angiogram of the thoracic and abdominal aorta was performed and the device was located in the visceral abdominal aorta. There was no compromise of flow noted to either the visceral vessels or distal aorta.

**Figure 3:**
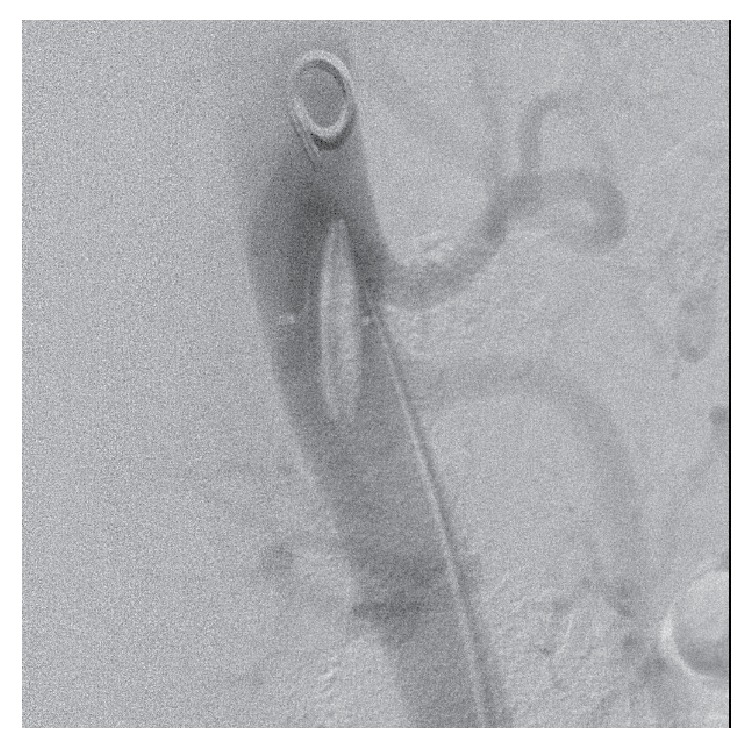
Digital subtraction angiography confirming embolized patent foramen ovale occlusion device within abdominal aorta.

**Figure 4:**
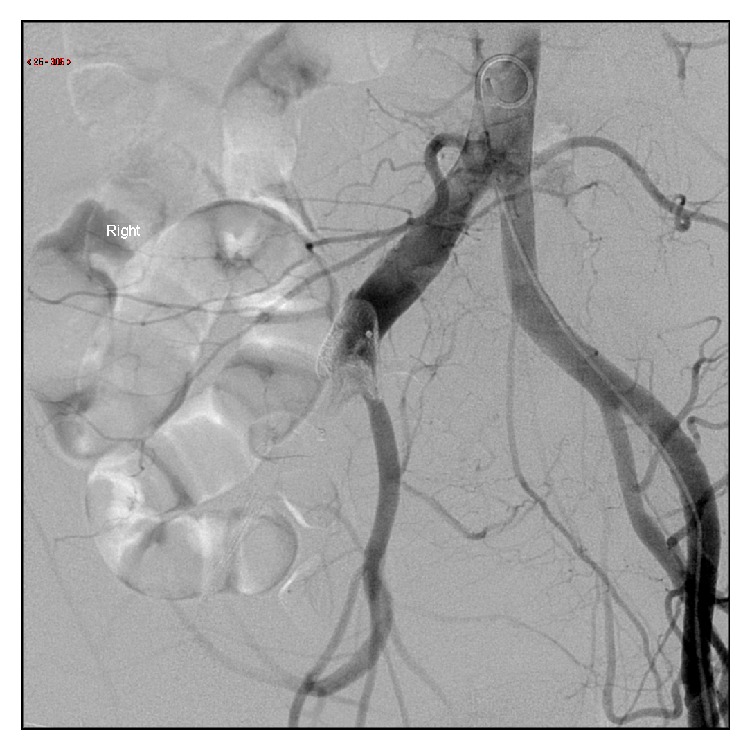
Digital subtraction angiography demonstrating distorted patent foramen ovale occlusion device in right common iliac artery.

Arrangements were then made for percutaneous device retrieval. The right common femoral artery was exposed using a transverse incision and a 20 Fr Cook sheath was advanced through the right common femoral artery. Heparin was administered intravenously. The device was located in the abdominal aorta (Figure 3) with no compromise of mesenteric vessels observed. The device was initially snared with a 20 mm gooseneck snare; however the device could not be retrieved into the sheath due to lack of compressibility when withdrawal into the 20 Fr sheath was attempted. The gooseneck snare was then substituted for an ANL retriever; despite multiple attempts the device would not deform to allow passage into the 20 F sheath (Figure 4). The device was partially distorted and could be retracted into the right common iliac artery. Once positioned into the common iliac artery, the patient underwent open retrieval via a right sided Rutherford Morris incision.

Peripheral pulses were present at the end of the retrieval and no distal angiogram was performed. However, the postsurgical course was complicated by bilateral lower limb claudication. This was the result of nonocclusive embolism to the tibial vessels requiring surgical thrombectomy after attempted percutaneous aspiration. No metal was present in the retrieved tissue which was thought to be chronic thrombus and fibrin detached off the device during attempted withdrawal into the sheath.

The patient was subsequently commenced on apixaban with no recurrent thromboembolic complications during 12 months' follow-up.

## 3. Discussion

Percutaneous device closure of the patent foramen ovale represents a therapeutic option for prevention of paradoxical emboli when considering the aetiology of cryptogenic stroke. Orthopnoea-platypnoea [7], decompression illness [8], and migraine [9, 10] have also been treated with this percutaneous technique. Procedural complications are uncommon, with PFO closure considered less technically challenging when compared to atrial septal defect closure. While complications such as cardiac perforation, arrhythmia, and air embolization are common to both PFO and ASD device closure, device embolism is rare in PFO closure cases. Imaging of the tissue margins in ASD closure is essential for device sizing and placement; when an ASD has inadequate tissue rims, device positioning may be problematic with an accompanying risk of device embolization. In addition, the desire to avoid device oversizing to prevent the complications related to device erosion may result in selecting a smaller device than necessary which may also predispose to device embolization. These issues are specific to ASD closure and are therefore not encountered in PFO closure.

When septal occlusion device embolization is encountered following percutaneous ASD closure, a number of strategies to facilitate percutaneous retrieval are recognized [11]. The use of a large caliber vascular sheath to allow device removal is recommended; creating a notch in the sheath tip may further facilitate device retraction into the vascular sheath. In order to appropriately compress the device and retract it into the sheath, retrieval requires snaring of the locking mechanism on the right atrial disc. This can be achieved typically with a gooseneck snare [12]. Difficulty may be encountered when the device embolizes into the left heart, as negotiating the aortic and mitral valves may be problematic. In this case, despite snaring the device appropriately in the abdominal aorta, it could not be retrieved into a large caliber sheath due to inability of the device to deform, likely reflecting the late timing of retrieval attempt. Furthermore, the lack of ability to deform the device may predispose to device fracture and contribute to distal embolization as encountered in this example.

The mechanism of device embolization is unclear. The device embolized into the left heart, the Amplatzer 30 mm fenestrated atrial septal defect occluder, has a 30 mm disc on the right atrial aspect, compared to 25 mm on the Amplatzer 35 mm PFO occlusion device. The use of the fenestrated ASD device in this case should theoretically reduce the risk of device embolization into the left heart. The device position was confirmed prior to release with the device advanced and then retracted while maintained on the delivery cable; it is possible that the device was partially dislodged at this stage. It is fortuitous that there was no left heart outflow obstruction or features of arrhythmia noted with device embolization.

## 4. Conclusions

Percutaneous PFO closure is an important therapeutic option in the treatment of cryptogenic stroke. Procedural complications are fortunately rare. This case highlights that the possibility of device embolization is not limited to ASD device closure, noting that no cases of device embolization were reported in the PROTECT trial. Importantly, in cases of late recognition of device embolization, the operator should be aware of the inability of these devices to retract and compress as they would when initially deployed which has implications for the potential for percutaneous retrieval.
